# 17β-Estradiol Regulates Glucose Metabolism and Insulin Secretion in Rat Islet β Cells Through GPER and Akt/mTOR/GLUT2 Pathway

**DOI:** 10.3389/fendo.2019.00531

**Published:** 2019-08-06

**Authors:** Che Bian, Bowen Bai, Qian Gao, Siyi Li, Yuyan Zhao

**Affiliations:** ^1^Department of Endocrinology, The First Affiliated Hospital of China Medical University, Shenyang, China; ^2^Department of Endocrinology and Metabolism, The Fourth Affiliated Hospital of China Medical University, Shenyang, China

**Keywords:** 17β-estradiol, islet β cells, type 2 diabetes mellitus, glucose transporter 2, insulin secretion

## Abstract

**Aims:** To explore the molecular mechanism by which 17β-estradiol (estrogen 2, E2) regulates glucose transporter 2 (GLUT2) and insulin secretion in islet β cells through G protein-coupled estrogen receptor (GPER) via Akt/mTOR pathway.

**Methods:** SPF-grade SD male rats were used to establish an *in vivo* type 2 diabetes model treated with E2. Rat insulinoma cells (INS-1) were cultured in normal or high glucose media with or without E2. Immunofluorescence double staining was used to detect GPER, GLUT2, insulin, and glucagon immunolocalization in rat islet tissues. Western blot was used to detect GPER, Akt, mTOR, and GLUT2 protein immunocontent. Real-time PCR detected Slc2a2 and glucose kinase (GK) content, and ELISA was used to detect insulin levels. Glucose uptake, GK activity and pyruvate dehydrogenase (PDH) activity were analyzed with glucose detection, GK activity and PDH activity assay kit.

**Results:** Immunofluorescence double staining confocal indicated that E2 treatment up-regulated expression levels of GPER, GLUT2, and insulin, while down-regulated glucagon. Western blot results revealed E2 increased GPER, Akt/mTOR pathway, and GLUT2 protein immunocontent. Real-time PCR showed E2 elevated Slc2a2, GK content. Moreover, E2 improved insulin secretion, glucose uptake, GK activity, and PDH activity.

**Conclusion:** Our findings indicated that exogenous E2 up-regulated GPER via the Akt/mTOR pathway to increase GLUT2 protein content and insulin secretion in islet β cells.

## Introduction

Type 2 diabetes mellitus (T2DM) is a chronic progressive disease with high morbidity and mortality that are of increasing concern (1). The prevalence of T2DM significantly increases in postmenopausal women (2), which indicates that the prevalence may be related to a decrease of estrogen.

Estrogen, of which the main active form is 17-estradiol (Estrogen 2, E2), primarily originates in the ovary and plays a wide range of physiological roles in the reproductive, cardiovascular, nervous and immune systems via combination with estrogen receptors (3). Previous studies have examined estrogen nuclear receptor α (ERα) and estrogen nuclear receptor β (ERβ) in-depth which were found to have an important effect on cell proliferation and apoptosis (4). In recent research, extensive attention has been given to one estrogen membrane receptor called G-coupled estrogen receptor (GPER). GPER is distributed throughout human tissues, especially in rat and mouse islet β cells (5). The recent research shows that E2 preserves functional β-cell mass and affects insulin secretion in islet β cells via GPER (6, 7). There are also studies which demonstrated E2 and GPER agonist improves insulin secretion on human and mice islets via GPER (8). However, no studies have confirmed whether E2 makes an action in promoting insulin secretion through glucose transporter 2 (GLUT2).

Glucose transporter 2 (GLUT2) is widely distributed in the liver, kidney, intestinal and islet tissues and is an important factor in the regulation of glucose metabolism in islet β cells. GLUT2 combines with the glucose kinase (GK) as the glucose receptor on islet β cells and increases glucose uptake to promote insulin secretion by sensing hyperglycemia. Pyruvate dehydrogenase (PDH) is a key enzyme in this process.

E2 binds to GPER and affects downstream metabolites through multiple pathways, including the PI3K/Akt pathway (9). Mammalian target of rapamycin (mTOR) is a serine threonine protein kinase with a molecular weight of 289 kd, which maintains blood glucose levels by regulating glucose uptake in peripheral tissues such as skeletal muscle and fat (10).

According to this context, we established a T2DM rat model *in vivo* and cultured high-glucose INS-1 cells *in vitro*, to detect changes to glucose metabolism and insulin secretion as well as the expression levels of relevant factors employing pretreatment with E2, GPER antagonist (G15), Akt inhibitor (PF 04691502), and mTOR inhibitor (rapamycin) to explore the possible molecular mechanism of GPER via Akt/mTOR pathway in islet β cells.

## Materials and Methods

### Animal Modeling

Male Sprague-Dawley (SD) rats (5 weeks, SPF grade, 150–180 g, a total of 24 rats) were obtained from Beijing Vital River Laboratory Animal Technology. The rats were fed in an SPF laboratory maintained at 22 ± 3°C at a humidity of 55 ± 5% with a cycle 12 h light/12 h dark (lights on at 0600)as well as free access to water and food. All animal experiments were approved by the Institutional Animal Care and Use Committee (IACUC) of China Medical University (Ethic No. 17012).

After adaptive feeding for 7 days, the rats were randomly divided into three groups as follows: (i) The type 2 diabetes mellitus drugs-treatment group (TD group, *n* = 8 rats): fed with high-fat, high-sugar diet (20% protein, 45% fat, and 35% carbohydrate, D12451, Beijing Huafu Kang Co., Ltd.) for 8 weeks (11). Rats were then injected intraperitoneally with 20 mg/kg streptozotocin (STZ, Sigma-Aldrich, Merck KGaA, Germany) Day 1 to Day 3 in the 9th week. STZ dissolves in citric acid and sodium citrate solution (12). Two weeks after STZ injection, E2 (Sigma-Aldrich, Merck KGaA, Germany) was injected intraperitoneally at 10 μg/kg for 8 weeks. E2 dissolves in olive oil. (ii) The type 2 diabetes mellitus group (TM group, *n* = 8 rats): the same diet and STZ injection as the TD group. Two weeks after STZ injection, rats were injected with isodose olive oil as an E2 control. The induction success rate for hyperglycemia in both TD and TM groups was 100% and no rats were lost. (iii) The normal control group (NC group, *n* = 8 rats): fed with control diet (28% protein, 12% fat, and 60% carbohydrate, Animal Center of China Medical University). Rats were injected with citric, sodium citrate solution, and isodose olive oil as a control for STZ and E2.

All animals fasted overnight and then took a 2 g/kg glucose oral glucose tolerance test (OGTT) and insulin release test, independently, 6 days and 10 weeks after STZ injection (13). The establishment of this model was evaluated by random blood glucose levels of rat tail vein >16.7 mmol/L (14) or fasting blood glucose levels >11.1 mmol/L (15). The end point of the experiment was a week after the last OGTT. After application of isoflurane anesthesia, the body weight and fat of rats were measured and then islet tissues were collected after cardiac perfusion by cold normal saline and preserved in 4% paraformaldehyde solution at 4°C for immunofluorescence, liquid nitrogen at −196°C for western blot and non-frozen tissue stored in RNA preservation fluid at −20°C for real-time PCR (16).

### Cell Culture

The rat insulinoma cell line (INS-1) was purchased from China Infrastructure of Cell Line Resources (Beijing, China). The cells were concomitantly incubated in RPMI-1640 medium with 10% fetal bovine serum (FBS, Gibco^TM^, Thermo Fisher Scientific, USA) and 3.4 μl/L β-mercaptoethanol (Sigma-Aldrich, Merck KGaA, Germany) in a humidified atmosphere containing 5% CO_2_ at 37°C. After cell density reached 80% once every 2 days, cells were passaged. After incubation in an FBS-free medium for 24 h at normal and high glucose with or without E2 and inhibitors, INS-1 cells were divided into the following groups (i) NG: normal glucose (5 mmol/L) for 24 h, (ii) NE: normal glucose + 0.1 μM E2 for 24 h, (iii) NGI: normal glucose + 15 μM G15 (Tocris Bioscience Minneapolis, USA) for 24 h, (iv) NAI: normal glucose + 1 μM Akt inhibitor (PF-04691502, Selleck Company, USA) for 24 h, (v) NMI: normal glucose + 10 μM mTOR inhibitor (rapamycin, Selleck Company, USA) for 48 h, (vi) HG: high glucose (30 mmol/L) for 24 h (17), (vii) HE: high glucose + 0.1 μM E2 for 24 h, (viii) HGI: high glucose + 15 μM G15 for 24 h, (ix) HAI: high glucose + 1 μM Akt inhibitor for 24 h, (x) HMI: high glucose + 10 μM mTOR inhibitor for 48 h (18).

### Immunofluorescence

In order to study the pancrease morphology, 8 pancreases from each group of rats were removed and weighed, excised fat and lymphatic tissues for serial section for serial section (19, 20). β-cell mass was measured by point-counting stereology according to the previous research (21). After fixing in cold acetone, tissues were incubated with primary antibodies (GPER, ab39742; GLUT2, ab54460, insulin, ab181547; glucagon, ab10988; 1: 200, respectively, Abcam, USA) at 4°C overnight. Then tissues were washed and incubated with the secondary antibody (Alexa Fluor^®^ 488 Conjugate anti-rabbit IgG and Alexa Fluor^®^ 555 Conjugate anti-rabbit IgG, 1:1,000, #4412 and #4413, Cell Signaling Technology, USA) at room temperature for 1 h. Sections were washed again and then DAPI stained (#4083, Cell Signaling Technology, USA) at room temperature for 10 min. Images of the sections were taken under an Olympus fluorescence confocal microscope (FV-1000, Japan).

### Western Blot

Islets were isolated with collagenase (C0130 Sigma-Aldrich, Merck KGaA, Germany) as described in previous literature (22, 23). Rat islet tissues and cell samples were extracted with lysis buffer (P0013B, Beyotime Biotechnology, China) containing 1% Triton X-100, 1% deoxycholate, 0.1% SDS, phosphatase Inhibitor cocktail and protease Inhibitor cocktail (78420 and 78429, Thermo Fisher Scientific, USA). After centrifugation at 16,000 × g for 20 min, a BCA protein assay kit (P0010S, Beyotime Biotechnology, China) was used to measure protein concentration. Protein bands were separated by 6% and 10% SDS-PAGE electrophoresis and then transferred onto PVDF membranes (0.45 mm, Millipore, USA) for 1 h to detect GPER, p-Akt, t-Akt, GLUT2 and β-actin, as well as 3 h for p-mTOR and t-mTOR. The membranes were cut based on the molecular weight of target proteins after transfer and then blocked with 5% BSA for 1 h. Membranes were incubated with primary antibodies (GPER, SAB2107697; GLUT2, AV41706; β-actin, SAB2100037; 1:1,000, respectively, Sigma-Aldrich, Merck KGaA, Germany; p-Akt, #5012; t-Akt, #4691; p-mTOR, #5536; t-mTOR, #2983; 1:1,000, respectively, Cell Signaling Technology, USA) at 4°C overnight, and then secondary antibody (Anti-rabbit IgG-HRP, 1:2,000, #7074, Cell Signaling Technology, USA) at room temperature for 2 h after washed 3 times. Protein bands were measured with Pierce^TM^ ECL Plus Substrate (32109, Thermo Fisher Scientific, USA) in the MicroChemi 4.2 system (Jerusalem, Israel). Based on the similar molecular weight of target proteins, the primary and secondary antibodies were removed by stripping buffer (P0025N, Beyotime Biotechnology, China) and then the membranes were blocked and the antibodies were incubated for subsequent experiments.

### Real-time PCR

Total RNAs were extracted with TRIzol reagent (Invitrogen, Thermo Fisher Scientific, USA) according to the manufacturer's instructions. Primer sequences were shown in Table 1 (Sangon Biotech, China). PrimeScript™ RT Reagent Kit with gDNA Eraser (Perfect Real Time), TB Green™ Premix Ex Taq™ II (Tli RNase H Plus) and the Thermal Cycler Dice Real-time PCR system (TaKaRa, Japan) of 1,000 ng total RNA were used for each reaction.

**Table 1:** Primer sequences.

**Primers**	**Genbank accession number**	**Sequences**	**Length (bp)**	**Tm (^**°**^C)**
Slc2a2-forward	NM_012879	5′-CAC CAG CAC ATA CGA CAC CAG AC-3′	23	63.5
Slc2a2-reverse		5′-TGG ACA CAG ACA GAG ACC AGA GC-3′	23	63.8
GK-forward	NM_024381	5′- GTG AGG CAC GAA GAC CTA GAC AAG-3′	24	62.9
GK-reverse		5′- TCA CCA TTG CCA CAT CCA TC-3′	20	58.2
β-actin-forward	NM_031144	5′-CAC TAT CGG CAA TGA GCG GTT CC−3′	23	64.0
β-actin-reverse		5′-CAG CAC TGT GTT GGC ATA GAG GTC−3′	24	63.7

### Measurement of Glucose Uptake

Glucose uptake was determined by a glucose uptake colorimetric assay kit (ab136955, Abcam, Cambridge, MA, USA) according to the manufacturer's instruction. Briefly, INS-1 cells were starved in a 96-well plate with FBS-free 1640 medium overnight and then incubated with Krebs-Ringer-Phosphate-Hepes (KRPH) buffer (20 mM HEPES, 5 mM KH_2_PO_4_, 1 mM MgSO_4_, 1 mM CaCl_2_, 136 mM NaCl, 4.7 mM KCl, pH 7.4) containing 2% BSA for 40 min. Then cells were, respectively, incubated in normal or high glucose medium and pre-treated in different groups (no treatment, 0.1 μM E2 for 30 min, 15 μM G15 for 30 min, 1 μM PF-04691502 for 30 min and 10 μM rapamycin for 1 h). Finally, 10 μl of 2-deoxy-glucose (2-DG) was added for a 20 min incubation, then the substrate oxidation reaction was detected by OD at wavelength 412 nm.

### Glucokinase Activity and Insulin Secretion

INS-1 cells cultured in normal or high glucose medium were pretreated with either no treatment, 0.1 μM E2 for 30 min, 15 μM G15 for 30 min, 1 μM PF-04691502 for 30 min or 10 μM rapamycin for 1 h, under the same concentration as the measurement of glucose uptake. GK activity was determined by the rat glucokinase enzyme linked immunosorbent assay (ELISA) kit (SEA667Ra, Cloud-Clone Corp., USA). Serum samples collected by insulin release testing after STZ injection 2 and 10 weeks and cell supernatant after 24 h incubated with normal or high glucose were analyzed with the rat insulin ELISA kit (CEA448Ra, Cloud-Clone Corp., USA). OD_450_ value was measured with Power Wave XS (Biotek, USA).

### Pyruvate Dehydrogenase Activity

INS-1 cells cultured in normal or high glucose medium were pretreated with either no treatment or 0.1μM E2 for 30 min, PDH activity was measured by a Pyruvate Dehydrogenase (PDH) Enzyme Activity Microplate Assay Kit (ab109902, Abcam, USA).

### Statistical Analysis

All measurements were repeated three times. Results were expressed as the mean ± standard deviation. Comparison of means was completed with one-way ANOVA after the data were submitted to normality and homoscedasticity tests. *P* < 0.05 was considered to be statistically significant. Statistical analysis was performed using SPSS 22.0 statistical software (SPSS Inc., USA).

## Results

### Immunolocalization of GPER and GLUT2 in Rat Islet Tissues

**Figure 1:**
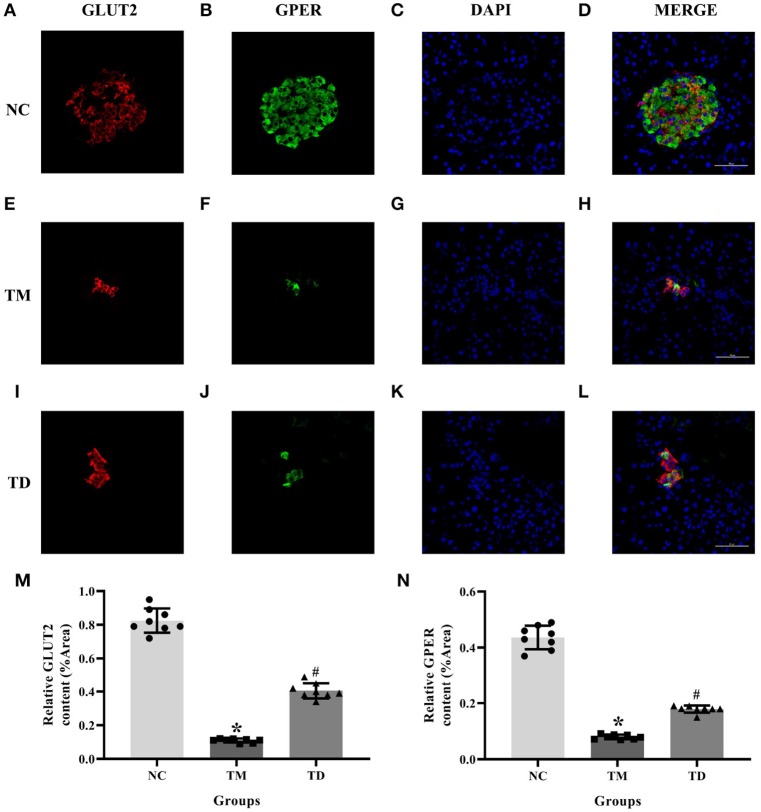
Immunolocalization of GPER and GLUT2 in rat islet tissues. **(A–D)** NC group, **(E–H)** TM group, and **(I–L)** TD group. **(M,N)** Relative GLUT2 and GPER content. Immunofluorescence double staining confocal of rat islet β cells for GPER (green), GLUT2 (red). Nuclei were stained with DAPI (blue) and merged images. Scale bars: 50 μm. GPER, G protein-coupled estrogen receptor; GLUT2, Glucose transporter 2; DAPI, 4′, 6-diamidino-2-phenylindole; NC, the normal control group; TM, the type 2 diabetes mellitus group; TD, the type 2 diabetes mellitus drug-treatment group. *n* = 8 rats in each group. Results of one-way ANOVA demonstrated *P < 0.05 compared to the NC group. ^#^*P* < 0.05 compared to the TM group. Data were presented as the mean ± standard deviation.

We adopted immunofluorescence double staining confocal detection to explore the immunolocalization levels of GPER and GLUT2 in rat islet tissues. Our results showed that the immunolocalization levels of GPER and GLUT2 in rat islet tissues were significantly more decreased in the TM group than those of the NC group. Moreover, the immunolocalization levels of GPER and GLUT2 in the TD group were significantly increased compared with the TM group (*P* < 0.05), as shown in Figure 1.

### Immunolocalization of Insulin and Glucagon in Rat Islet Tissues

**Figure 2:**
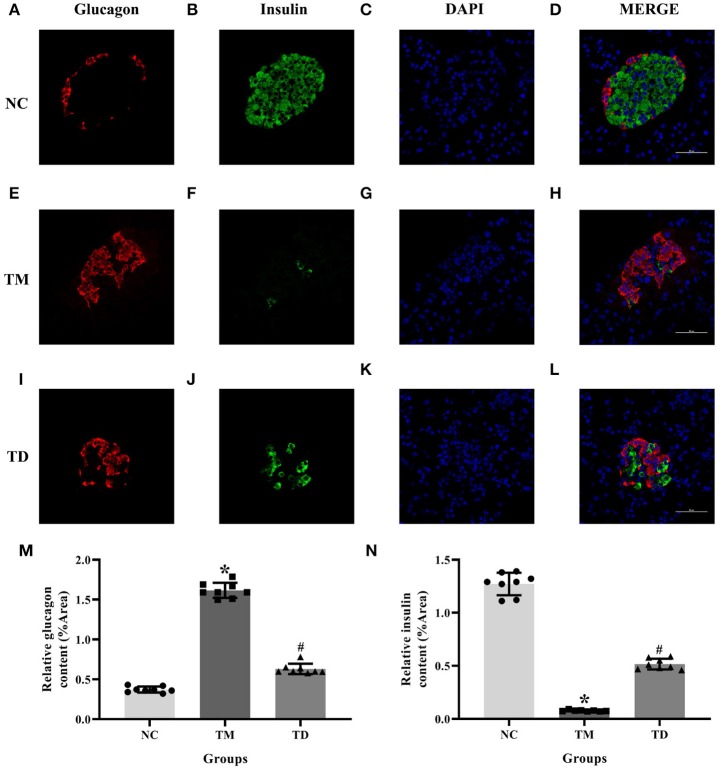
Immunolocalization of insulin and glucagon in rat islet tissues. **(A–D)** NC group; **(E–H)** TM group; and **(I–L)** TD group. **(M,N)** Relative GLUT2 and GPER content. Immunofluorescence double staining confocal of rat islet β cells for glucagon (red), insulin (green). Nuclei were stained with DAPI (blue) and merged images. Scale bars: 50 μm. DAPI, 4′, 6-diamidino-2-phenylindole; NC, the normal control group; TM, the type 2 diabetes mellitus group; TD, the type 2 diabetes mellitus drug-treatment group. *n* = 8 rats in each group. Results of one-way ANOVA demonstrated *P < 0.05 compared to the NC group. ^#^*P* < 0.05 compared to the TM group. Data were presented as the mean ± standard deviation.

Serial sections immunofluorescence double staining was applied to immunolocalize insulin and glucagon in rat islet tissues. Results indicated that the immunolocalization level of insulin was much more declined in the TM group than that of the NC group. Moreover, the immunolocalization level of insulin in the TD group was more obviously elevated than that of the TM group. The immunolocalization level of glucagon was more elevated in the TM group than that of the NC group and declined in the TD group compared with the TM group (*P* < 0.05), as shown in Figure 2.

### Effects of E2 on Physiological Indexes and Biofactors in Rats

OGTT showed that glucose levels in the TM groups at 6d and 10w had more significant rises compared with the NC group. Moreover, glucose levels in the TM 10w group were higher than that of TM 6d and TD groups. The insulin levels in TM 6d were elevated, while reduced in TM10w compared with the NC group. The insulin levels in the TD group were more higher than that of the TM 10w group (*P* < 0.05).

The contents of Slc2a2 and GK mRNA, and the protein of GPER and GLUT2 in rat islet tissues were detected by real-time PCR and western blot. The contents of Slc2a2, GK mRNA and GPER, GLUT2 protein were reduced in the TM group, while they were elevated in the TD group (*P* < 0.05).

**Figure 3:**
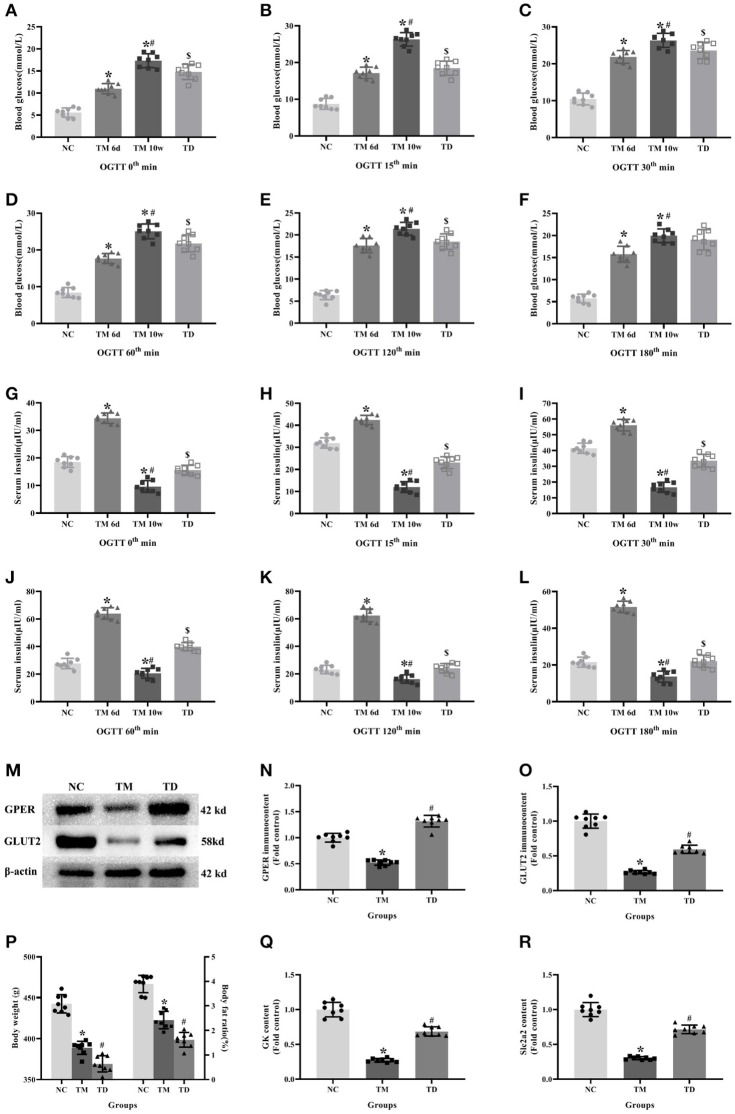
Effects of E2 on physiological indexes and biofactors in rats. **(A–L)** Blood glucose and insulin levels at different time point in OGTT. **(M)** Protein bands. **(N,O)** GPER and GLUT2 immunocontents, normalized to the internal control (β-actin). **(P)** Body weight and body fat ratios. **(Q,R)** Slc2a2 and GK contents. NC, the normal control group; TM 6d, the type 2 diabetes mellitus group 2 weeks after STZ injection; TM 10w, the type 2 diabetes mellitus group 10 weeks after STZ injection; TD, the type 2 diabetes mellitus drug-treatment group. **(A–L,P)** *n* = 8 rats in each group. **(M–O,Q,R)**. *n* = 8 rats in each group. *n* = randomly 3/8 rats in each group. Results of one-way ANOVA demonstrated *P < 0.05 compared to the NC group. ^#^*P* < 0.05 compared to the TM 6d or TM groups. ^$^*P* < 0.05 compared to the TM 10w group. Data were presented as the mean ± standard deviation.

Body weight and body fat ratios were evidently declined in the TM group than those in the NC group. However, they were risen in the TM group than those in the TD group (*P* < 0.05; Figure 3).

### Glucose Metabolism and Insulin Secretion in INS-1 Cells

**Figure 4:**
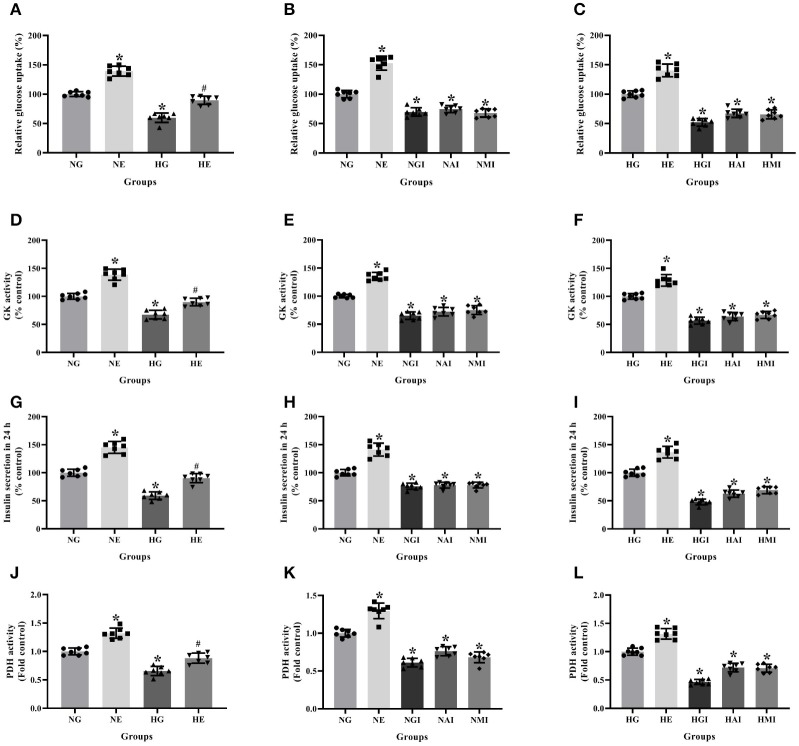
Glucose metabolism and insulin secretion in INS-1 cells. Glucose uptake in **(A)** NG and HG groups with or without E2 treatment, **(B)** NG groups with different treatment, and **(C)** HG groups with different treatment. GK activity in **(D)** NG and HG groups with or without E2 treatment, **(E)** NG groups with different treatment and **(F)** HG groups with different treatment. Twenty four hours insulin secretion in **(G)** NG and HG groups with or without E2 treatment, **(H)** NG groups with different treatment and **(I)** in HG groups with different treatment. PDH activity in **(J)** NG and HG groups with or without E2 treatment, **(K)** NG groups with different treatment and **(L)** HG groups with different treatment. NG, normal glucose; NE, normal glucose + E2; NGI, normal glucose + GPER antagonist; NAI, normal glucose + Akt inhibitor; NMI, normal glucose + mTOR inhibitor; HG, high glucose; HE, high glucose + E2; HGI, high glucose + GPER antagonist; HAI, high glucose + Akt inhibitor; HMI, high glucose + mTOR inhibitor. *n* = 7 in each group. Results of one-way ANOVA demonstrated *P < 0.05 compared to the NG or HG group. ^#^*P* < 0.05 compared to the HG group. Data were presented as the mean ± standard deviation.

Glucose uptake, GK activity, PDH activity and insulin secretion were measured to explore glucose metabolism in INS-1 cells cultured in normal or high glucose medium and pretreated respectively with or without E2, G15, PF-04691502 and rapamycin. Glucose uptake, GK activity, PDH activity and insulin secretion were reduced in the HG group compared with the NG group. It was clear that E2 increased glucose uptake, GK activity, PDH activity and insulin secretion while G15, PF-04691502, and rapamycin decreased these functions in both cultures (*P* < 0.05; Figure 4).

### Slc2a2 and GK Content in INS-1 Cells

**Figure 5:**
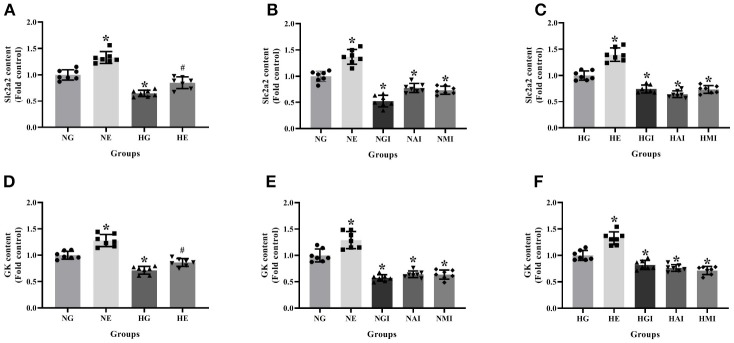
Slc2a2 and GK content in INS-1 cells. Slc2a2 content in **(A)** NG and HG groups with or without E2 treatment, **(B)** NG groups with different treatment and **(C)** HG groups with different treatment. GK content in **(D)** NG and HG groups with or without E2 treatment, **(E)** NG groups with different treatment, and **(F)** HG groups with different treatment. NG, normal glucose; NE, normal glucose + E2; NGI, normal glucose + GPER antagonist; NAI, normal glucose + Akt inhibitor; NMI, normal glucose + mTOR inhibitor; HG, high glucose; HE, high glucose + E2; HGI, high glucose + GPER antagonist; HAI, high glucose + Akt inhibitor; HMI, high glucose + mTOR inhibitor. *n* = 7 in each group. Results of one-way ANOVA demonstrated *P < 0.05 compared to the NG group. ^#^*P* < 0.05 compared to the HG group. Data were presented as the mean ± standard deviation.

Real-time PCR was employed to explore Slc2a2 and GK mRNA contents cultured in normal or high glucose medium and pretreated, respectively, with E2, G15, PF-04691502, and rapamycin. Slc2a2 and GK mRNA contents were declined in the HG group compared with the NG group. It was observed that E2 increased Slc2a2 and GK mRNA content while G15, PF-04691502 and rapamycin decreased these mRNA content in both cultures (*P* < 0.05; Figure 5).

### Protein Immunocontent in Normal and High Glucose Cultured INS-1 Cells

**Figure 6:**
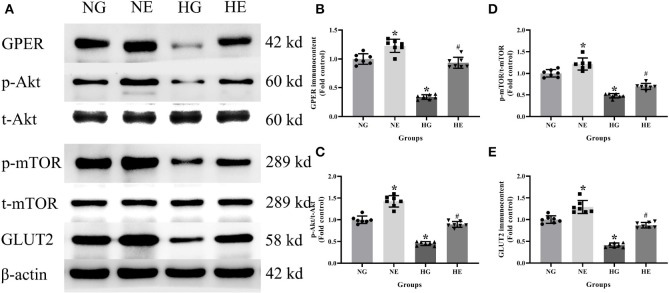
Protein immunocontent in normal and high glucose cultured INS-1 cells with or without E2 treatment. **(A)** Protein bands; **(B)** GPER immunocontent, normalized to the internal control (β-actin); **(C)** p-Akt/t-Akt; **(D)** p-mTOR/t-mTOR; and **(E)** GLUT2 immunocontent, normalized to the internal control (β-actin). NG, normal glucose; HG, high glucose; NE, normal glucose pre-treated with E2; HE, high glucose pre-treated with E2. *n* = 7 in each group. Results of one-way ANOVA demonstrated *P < 0.05 compared to the NG group. ^#^*P* < 0.05 compared to the HG group. Data were presented as the mean ± standard deviation.

Western blot was used to detect GPER, p-Akt/t-Akt, p-mTOR/t-mTOR, and GLUT2 protein immunocontents from cells cultured in normal or high glucose medium. It was evident that compared with the NG group, the GPER, p-Akt/t-Akt, p-mTOR/t- mTOR, and GLUT2 protein immunocontents were reduced in the HG group while increased in the NE group. Moreover, the protein immunocontents were elevated in the HE group than that of the HG group (*P* < 0.05; Figure 6).

### Protein Immunocontent in Normal Glucose Cultured INS-1 Cells With Different Treatment

**Figure 7:**
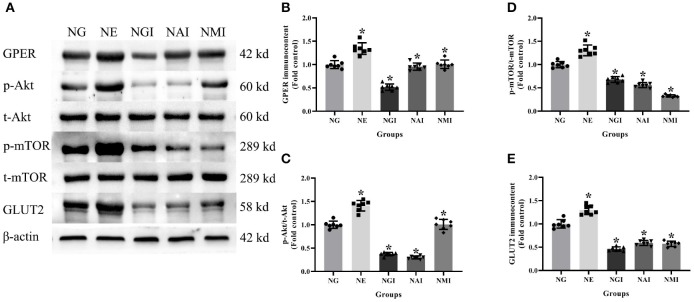
Protein immunocontent in normal glucose cultured INS-1 cells with different treatment. **(A)** Protein bands; **(B)** GPER immunocontent, normalized to the internal control (β-actin); **(C)** p-Akt/t-Akt; **(D)** p-mTOR/t-mTOR; and **(E)** GLUT2 immunocontent in NG groups with different treatment, normalized to the internal control (β-actin). NG, normal glucose; NE, normal glucose + E2; NGI, normal glucose + GPER antagonist; NAI, normal glucose + Akt inhibitor; NMI, normal glucose + mTOR inhibitor. *n* = 7 in each group. Results of one-way ANOVA demonstrated *P < 0.05 compared to the NG group. Data were presented as the mean ± standard deviation.

**Figure 8:**
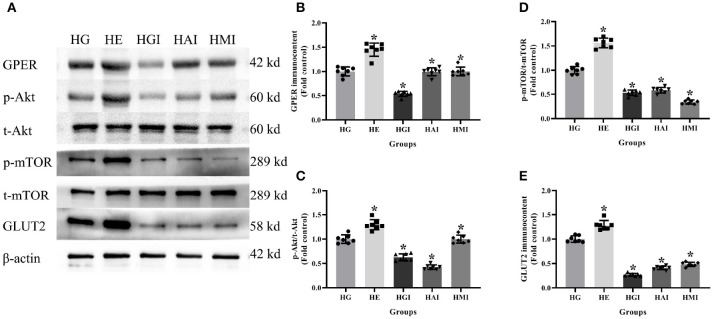
Protein immunocontent from INS-1 cells cultured in high glucose with different treatments. **(A)** Protein bands; **(B)** GPER content, normalized to the internal control (β-actin); **(C)** p-Akt/t-Akt; **(D)** p-mTOR/t-mTOR; and **(E)** GLUT2 immunocontent in HG groups with different treatments, normalized to the internal control (β-actin). HG, high glucose; HE, high glucose + E2; HGI, high glucose + GPER antagonist; HAI, high glucose + Akt inhibitor; HMI, high glucose + mTOR inhibitor. *n* = 7 in each group. Results of one-way ANOVA demonstrated *P < 0.05 compared to the HG group. Data were presented as the mean ± standard deviation.

In order to explore whether E2 influences GLUT2 protein content through Akt/mTOR pathway in normal or high glucose medium, western blot was applied to detect GPER, p-Akt/t-Akt, p-mTOR/t-mTOR, and GLUT2 protein immunocontents pretreated with E2, G15, PF-04691502, and rapamycin. E2 treatment was found to enhance GPER, p-Akt/t-Akt, p-mTOR/t-mTOR, and GLUT2 protein immunocontents while G15 suppressed such concentrations compared with the NG group (*P* < 0.05). It was observed that PF-04691502 inhibited p-Akt/t-Akt, p-mTOR/t-mTOR, and GLUT2 immunocontents (*P* < 0.05), however, it had no significant impact on GPER protein immunocontent (*P* > 0.05). Somewhat similarly, rapamycin inhibited p-mTOR/t-mTOR and GLUT2 content (*P* < 0.05), while it had no distinct effect on GPER and p-Akt/t-Akt protein content (*P* > 0.05; Figures 7, 8).

## Discussion

The incidence of type 2 diabetes mellitus (T2DM) has been increasing globally as a result of a combination of genetic and environmental factors (24). This disease has an insidious onset, longer duration, increasing rates of mortality and disability; it's mostly in adults, which draws much attention (25). The main pathophysiological changes associated with T2DM are β cell dysfunction and insulin resistance, while glucose metabolic disorders, elevated blood glucose levels, and damage to multiple organ systems are the main manifestations (26, 27). The pathogenesis of T2DM has not been fully elucidated. However, it has been reported that the abnormal structure and function of islet β cells has a close connection with the pathogenesis of T2DM (28, 29). Any breakdown of this pathogenetic chain can lead to the occurrence of the disease from the synthesis and secretion of insulin by islet β cells to the biological effects of binding of insulin to specific receptors (30).

Based on the above literature, we established a T2DM rat model *in vivo* in order to simulate type 2 diabetes pathogenesis in humans with a high-fat and high-glucose diet resulting in obesity, insulin resistance as well as STZ injection which may lose the compensation of islet β cells. The results of blood glucose, insulin levels and body fat ratio were constant with the T2DM patients reported in the literature (31). Previous literatures showed that body fat ratio were reduced and abnormal insulin secretion were improved in female SD rats treated with E2 after ovary resection operation (32), consistent with male SD rats in our results.

GLUT2 is required in response to glucose stimulation by islet β cells and is widely expressed in liver cells, islet cells, and epithelial cells with the absorption function of the small intestine and kidney (33). GLUT2 is one of the most important glucose transporters, whose function has been observed to be damaged and down-regulated expression among T2DM patients (34). Similarly, GK is a kind of hexokinase, which mainly exists in mature liver cells and islet β cells (35). GLUT2 acts as a glucose sensor in islet β cells along with GK to increase the intake of glucose for elevated blood glucose (36, 37). It also improves the activity of GK, while GK catalyzes glucose into glucose 6-phosphate to promote glycolysis, PDH activity, and the tricarboxylic acid cycle (38). This increases ATP formation, which leads to the closure of ATP-sensitive potassium channels, and the depolarization of the cell membrane as well as the internal flow of calcium ion, thus promoting insulin secretion (39).

Our research showed that Slc2a2, GK content and GLUT2 protein in the TM group was down-regulated, indicating that GLUT2 glucose transport and metabolic functions were damaged in T2DM rats, which is demonstrated in the supplemental results. INS-1 cells were cultured with normal and high glucose medium *in vitro*, and relevant tests were performed to explore the consistency of the subsequent results both *in vivo* and *in vitro*.

Our results showed that a high-glucose environment inhibited glucose uptake rates, GK activities and insulin secretion as well as Slc2a2, GK content, and GLUT2 protein, are consistent with our results *in vivo*. This finding indicates that hyperglycemia inhibits the GLUT2 perception, reduces glucose uptake and transport, as well as inhibits GK, PDH activity at the same time, which leads to a down-regulation of glucose uptake rate, obstructs glucose metabolism, therefore results in insufficient insulin secretion and further elevation of blood glucose.

PI3K/Akt signaling pathway was shown to be the key to obesity-induced insulin resistance and T2DM (40). Mammal rapamycin target protein (mTOR) is the downstream product of this signaling pathway, functioning as part of cell growth regulation and response to the cell nutrition state (41). Moreover, PI3K/Akt can affect islet β cell metabolism and apoptosis by regulating insulin-related signaling pathways, thus affecting insulin secretion and blood glucose regulation, which may play an important role in maintaining body metabolic homeostasis (42). To investigate the relationship between PI3K/Akt pathway and GLUT2-induced glucose metabolism, INS-1 cells cultured with normal glucose and high glucose were pretreated with Akt and mTOR inhibitors (PF-04691502, rapamycin), respectively. Furthermore, our results showed that high glucose inhibits the relative expression levels of p-Akt/t-Akt and p-mTOR/t-mTOR. Akt and mTOR inhibitors inhibited glucose uptake, GK activity and insulin secretion. A high glucose environment down-regulated the relative expression of GLUT2, GK mRNA, p-mTOR/t-mTOR and GLUT2 proteins in high and normal glucose cultured INS-1 cells. However, the relative expression of p-Akt/t-Akt was inhibited by Akt inhibitors rather than by mTOR inhibitors suggesting that high glucose down-regulates the expression of Akt/mTOR pathway. Moreover, Akt can regulate the expression of mTOR, affecting glucose transport uptake and insulin secretion in INS-1 cells cultured with both normal and high glucose.

Estrogen, whose most active form is E2, is a steroid hormone which mainly acts by combination with estrogen receptors (43). Previous literature has shown that the incidence of T2DM in postmenopausal women is significantly increased compared with premenopausal individuals (44). This suggests that a decrease in estrogen may be associated with the occurrence and development of T2DM. The classical estrogen receptors (ER) are nuclear estrogen receptor, including ERα and ERβ, which participate in the regulation of enormous complex physiological processes in humans (45). GPER is one of the membrane-bound estrogen receptors with rapid action and independence on gene regulation (46). Previous studies have indicated that E2 plays a neuroprotective role in activating ER and improving the efficiency of glucose transporters (47). Estrogen can also regulate glycolysis by promoting the expression of key enzymes in glycolysis, thus interfering glucose metabolism in tumor cells (48). Moreover, studies have suggested that GPER, widely distributed in a variety of tissues, including cytomembrane of islet β cells, both in rats and mice, can interact with the epidermal growth factor receptor (EGFR) -related signaling pathway and crosstalk with multiple other signaling pathways (49).

In order to explore whether E2 regulates glucose metabolism and insulin secretion via GPER and Akt/mTOR pathway, T2DM rats were treated with E2 and INS-1 cells in normal and high glucose cultures pretreated with E2 and G15, respectively. The previous studies showed that E2 promoted insulin secretion and reduced blood glucose levels in T2DM mice (8), and that E2 reduced glucagon and increased glucagon-like peptide for the balance of glucostasis (50). Besides, other studies indicated that E2 increases insulin and decreases glucagon in human pancreatic islets (6), consistent with our present study. In this study E2 was confirmed to increase insulin secretion and improve glucose metabolism, consistent with previous studies on the benefits of improvement on glucose metabolism treated with insulin (51). However, there is no literature supporting the E2 benefits instead of insulin therapy considering the possible side effects of E2 therapy in large samples of people (52). It will be an interesting topic on how to avoid the side effects of E2 in type 2 diabetes patients.

E2 was observed to up-regulate the content of Slc2a2 and GK, as well as elevate the levels of GPER and GLUT2 protein in INS-1 cells and TD group rats, which suggests that GPER is expressed in rat islet β cells and E2 can improve glucose metabolism and promote insulin secretion via GPER. The results of *in vitro* experiment showed that E2 increased glucose uptake, GK activity, PDH activity and insulin secretion and also up- regulated the relative expression of Slc2a2, GK content, GPER, p-Akt/t-Akt, p-mTOR/t-mTOR, and GLUT2 protein in normal and high-glucose cultures. In contrast, the G15 group was observed to have the opposite effect. These findings indicated that E2 can regulate the Akt/mTOR pathway by GPER to improve glucose metabolism and insulin secretion.

In sum, the treatment of T2DM with E2 improved glucose metabolism and insulin secretion via GPER. Further exogenous E2 eased glucose metabolism dysfunction in high-glucose cultured INS-1 cells through GPER and Akt/mTOR pathway, while G15 demonstrated the opposite effect. These results suggested that E2 can improve glucose metabolism and insulin secretion of islet β cells through GPER via Akt/mTOR pathway. These findings may provide a new theoretical basis for the treatment of T2DM.

## Data Availability

The datasets generated for this study are available on request to the corresponding author.

## Ethics Statement

The procedures for care and use of animals were approved by the Institutional Animal Care and Use Committee (IACUC) of the First Affiliated Hospital of China Medical University (Ethic No. 17012) and all experimental operations were complied with Guide for Laboratory Animal Care and Use and Animal Welfare Act. All applicable institutional and governmental regulations concerning the ethical use of animals were followed.

## Author Contributions

CB and YZ designed the experiments and wrote the manuscript. CB, BB, QG, and SL carried out the experiments. CB and BB collected and analyzed the data.

### Conflict of Interest Statement

The authors declare that the research was conducted in the absence of any commercial or financial relationships that could be construed as a potential conflict of interest.
